# Mechanical stress-induced STAT3/YAP signaling in fibroblasts programs immunosuppressive lymph node remodeling in lung squamous carcinoma

**DOI:** 10.7150/ijbs.132656

**Published:** 2026-06-17

**Authors:** Jie Cai, Zhao An, Wei Gao, Ryan Li, Zhe-Sheng Chen, An Li, Lei Zhu, Yingran Shen

**Affiliations:** 1 Department of Thoracic Surgery, Shanghai Pulmonary Hospital, School of Medicine, Tongji University, 507 Zhengmin Road, Shanghai 200433, China.; 2 Department of Radiation Oncology, Shanghai Fourth People's Hospital, School of Medicine, Tongji University, Shanghai, China.; 3 Department of Pharmaceutical Sciences, College of Pharmacy and Health Sciences, St. John's University, 8000 Utopia Parkway, Queens, New York 11439, USA.; 4 Department of Oncology, The First Affiliated Hospital of Gannan Medical University, Ganzhou, Jiangxi, China.

**Keywords:** lung squamous cell carcinoma, solid stress, lymph node, metastasis

## Abstract

Lung squamous cell carcinoma (LUSC) frequently metastasizes to lymph nodes, yet how tumor-intrinsic biomechanical cues influence nodal immune environments remains unclear. Here, we identify solid stress, generated during tumor expansion, as a key driver of pre-metastatic niche formation in tumor-draining lymph nodes (TDLNs). Using murine models with defined solid stress levels, we show that high-stress tumors induce early TDLN remodeling characterized by fibroblast activation and accumulation of immunosuppressive S100a8⁺ myeloid cells. Single-cell and spatial transcriptomics reveal that Col1a1⁺ fibroblasts upregulate STAT3 and YAP-dependent chemokines (CCL2, CSF1), establishing a fibroblast-myeloid signaling axis that supports myeloid recruitment and suppresses CD8⁺ T cell infiltration. Functional blockade of CCR2, CSF1R, STAT3, or YAP disrupts fibroblast-myeloid co-localization, restores antigen-presenting cell populations, and enhances CD8⁺ cytotoxic T cells activity, leading to reduced lymph node metastasis and improved survival. Mechanistically, chromatin accessibility and ChIP assays reveal direct binding of p-STAT3 and YAP at inflammatory enhancer elements in fibroblasts under mechanical strain. Cross-species spatial transcriptomic analysis confirms the inverse association between Col1a1⁺ fibroblast density and CD8A⁺ T cell presence in human LUSC tissues. Our findings uncover a mechano-immune circuit whereby tumor-derived solid stress activates fibroblast-STAT3/YAP signaling to remodel TDLNs into immunosuppressive, metastasis-permissive niches. Targeting this biomechanical axis offers a promising strategy to intercept early lymphatic dissemination in LUSC.

## Introduction

Lung squamous cell carcinoma (LUSC) accounts for 25-30% of non-small cell lung cancers and remains a major cause of cancer-related death 1-2. According to GLOBOCAN 2022, over 2.48 million new lung cancer cases and 1.8 million deaths occur annually 3. Unlike adenocarcinoma, LUSC lacks targetable mutations and typically presents with abundant stroma and immune evasion 4-5. Lymph node metastasis is a critical determinant of poor prognosis, but its underlying biology remains obscure 6-7. Emerging studies suggest that tumors may condition tumor-draining lymph nodes (TDLNs) before metastasis, forming a pre-metastatic niche. While this process has been described in other tumor types, its relevance in LUSC is poorly understood 8-10.

Recent work in breast and pancreatic cancers indicates that tumor-derived signals can reprogram lymph nodes by activating stromal fibroblasts and attracting suppressive myeloid cells 11-12. However, whether similar mechanisms apply to LUSC remains unclear. Moreover, the potential role of mechanical forces, particularly solid stress generated by tumor expansion, has not been adequately explored. Solid stress influences vasculature and fibroblast behavior in primary tumors, but its effect on distant immune environments is unknown 13-14. Studies suggest that biomechanical cues within the tumor microenvironment can influence immune regulation 15-16. Mechanical forces such as solid stress have been shown to affect stromal activation and immune cell recruitment in several cancer types 17-18. We hypothesized that LUSC-generated solid stress might remotely alter lymph node stromal-immune interactions. To test this, we established high- and low-stress murine models and applied single-cell, spatial, and immunological profiling to delineate stress-induced immune remodeling in TDLNs.

This study uncovers a biomechanical mechanism by which solid stress primes lymphatic tissues for metastasis. We show that stressed fibroblasts upregulate STAT3 and YAP-dependent chemokines, driving the accumulation of S100a8⁺ immunosuppressive myeloid cells and exclusion of CD8⁺ T cells, hallmarks of a pre-metastatic niche. These changes precede tumor cell invasion, indicating a preparatory phase for nodal colonization. Our findings identify TDLNs as active responders to tumor-derived stress and suggest that targeting fibroblast-myeloid crosstalk may delay early dissemination in LUSC.

## Materials and Methods

### Murine tumor models and solid stress measurement

C57BL/6 mice (6-8 weeks old) were subcutaneously injected with 1 × 10⁶ H520 or H1703 lung squamous cell carcinoma (LUSC) cells. Cells were resuspended in PBS and implanted using a 25-gauge needle. H520 tumors were classified as high solid stress (SS-high) and H1703 as low solid stress (SS-low) based on quantitative biomechanical measurements. Tumor growth was monitored twice weekly. Solid stress was quantified using planar cut relaxation and strain-induced deformation assays. For orthotopic studies, tumor fragments (1 mm³) from H520 and H1703 were implanted into the left lung lobe of anesthetized C57BL/6 mice. The procedure was performed using a sterile surgical technique under isoflurane anesthesia, with analgesia provided using buprenorphine post-surgery.

Solid stress was quantified *ex vivo* by two complementary methods. Planar cut relaxation was used to assess tissue retraction upon incision, where H520 tumors exhibited pronounced opening and deformation, indicating higher intrinsic stress. Strain-induced deformation assays were then performed to confirm these findings, demonstrating significantly greater residual strain release in H520 tumors than in H1703. These results established reproducible stress stratification across models. To address potential differences in solid stress classification between subcutaneous and orthotopic implantation models, we conducted additional experiments and have included a comparison of the stress classifications. For orthotopic studies, tumor fragments were implanted into the left lung lobe under anesthesia.

Specifically, for subcutaneous studies, tumors were implanted subcutaneously into the dorsal flanks of mice, whereas for orthotopic studies, tumor fragments were implanted into the left lung lobe under anesthesia. The two models were used in separate experimental sets to assess how solid stress classification remained consistent across implantation sites.

### Definition of high and low solid stress

High and low solid stress in the lung cancer cell lines (H520 and H1703) were defined based on the biomechanical measurements described above. High-stress tumors (H520) exhibited pronounced deformation and higher residual strain release, corresponding to greater tumor stiffness. In contrast, low-stress tumors (H1703) demonstrated minimal deformation under the same conditions, indicating a less rigid tumor structure.

### Lymph node analysis and flow cytometry

Axillary and brachial tumor-draining lymph nodes (TDLNs) were collected on days 7-21. For single-cell suspensions, the TDLNs were minced and enzymatically dissociated using a gentleMACS tissue dissociator (Miltenyi Biotec). Single-cell suspensions were stained with anti-CD11b, Ly6C, Ly6G, PD-L1, CD8, MHC-II, and FOXP3 antibodies and analyzed using a BD LSRFortessa flow cytometer with FlowJo software. Live/dead staining was performed using the Fixable Viability Dye (Thermo Fisher) to exclude dead cells from the analysis. S100a8⁺ myeloid cells were sorted for downstream assays.

### Single-cell and spatial transcriptomic profiling

TDLNs were processed using the 10x Genomics Chromium platform (3′ v3) and sequenced on an Illumina NovaSeq. The cells were encapsulated into nanoliter-sized droplets, and cDNA libraries were prepared according to the manufacturer's protocol. Data were analyzed with Cell Ranger and Seurat (v4.0) for clustering, UMAP visualization, pseudotime inference, and ligand-receptor interaction analysis (CellPhoneDB). Barcoded sections were aligned to the mm10 reference genome using Space Ranger, and gene expression was mapped to tissue structures. Co-localization analysis was performed to evaluate interactions between fibroblasts and myeloid cells in the lymph node microenvironment.

### Bone marrow chimeras

Recipient mice were lethally irradiated and reconstituted with GFP⁺ bone marrow. After eight weeks, tumors were implanted. To confirm the bone marrow origin of myeloid cells, GFP⁺CD11b⁺ cells from TDLNs were quantified by flow cytometry and immunofluorescence. Immunofluorescence staining was performed using anti-GFP and anti-CD11b antibodies (BioLegend), and images were obtained using a Leica SP8 confocal microscope.

### *In situ* staining and image analysis

LN cryosections were stained for COL1A1, S100a8, phosphorylated STAT3, YAP, and DAPI. Confocal imaging was performed using a Leica SP8 microscope. Spatial co-localization was quantified using ImageJ and CellProfiler.

### *Ex vivo* fibroblast mechanostimulation

Primary fibroblasts isolated from LNs were subjected to cyclic stretch (5%, 0.5 Hz, 24 h) on a Flexcell FX-5000 system. Fibroblasts were cultured in DMEM with 10% FBS and 1% penicillin/streptomycin (Gibco) prior to stimulation. Conditioned media from fibroblasts were applied to bone marrow-derived monocytes, and gene expression of S100a8, IL-10, and CD274 (PD-L1) was quantified by qPCR. Total RNA was isolated using the RNeasy Plus kit (Qiagen), and reverse transcription was performed with the High-Capacity cDNA Reverse Transcription Kit (Applied Biosystems). The qPCR analysis was performed using SYBR Green PCR Master Mix (Applied Biosystems).

### Pharmacologic interventions

Mice were treated by intraperitoneal injection for seven days with S3I-201 (STAT3 inhibitor), Verteporfin (YAP-TEAD blocker), PLX3397 (CSF1R inhibitor), or RS504393 (CCR2 antagonist). Dosing regimens were optimized through pilot toxicity assessments.

### ATAC-seq and ChIP-qPCR

FACS-sorted CD90⁺ fibroblasts were processed for ATAC-seq using the Omni ATAC protocol (Illumina). ATAC-seq libraries were constructed, amplified, and sequenced on an Illumina NovaSeq 6000 platform. Sequencing reads were aligned to the mm10 mouse genome and visualized using Integrative Genomics Viewer (IGV).

For ChIP-qPCR, antibodies against phospho-STAT3 and YAP were used to immunoprecipitate transcription factor-bound DNA. ChIP DNA was analyzed by qPCR to examine binding at regulatory elements near Ccl2 and Csf1. Enrichment values were normalized to input controls.

### Bulk RNA-seq and gene enrichment analysis

CD11b⁺S100a8⁺ and S100a8⁻ myeloid subsets were isolated by FACS and subjected to bulk RNA-seq. Differential gene expression was analyzed with DESeq2, and pathway enrichment was evaluated by GSEA using Hallmark and curated immune gene sets from MSigDB.

### T-cell cytotoxicity assays

CD8⁺ T cells purified from TDLNs were co-cultured with autologous tumor spheroids. Cytotoxicity was quantified by Annexin V/PI staining and lactate dehydrogenase (LDH) release assays.

### Statistical analysis

Data were analyzed using GraphPad Prism. Comparisons between two groups were performed using unpaired two-tailed t-tests, while comparisons involving more than two groups were analyzed using one-way ANOVA with Tukey's post-hoc test for multiple comparisons. Survival was assessed by Kaplan-Meier analysis and log-rank test. P value < 0.05 was considered statistically significant.

The sample sizes were determined based on preliminary power analyses, aiming for at least 80% statistical power to detect significant differences. The sample sizes used are indicated in the figure legends and were chosen to balance feasibility with adequate statistical power for detecting biologically relevant differences.

## Results

### Solid stress-induced lymph node remodeling precedes metastasis and reprograms myeloid immunity

**Figure 1:**
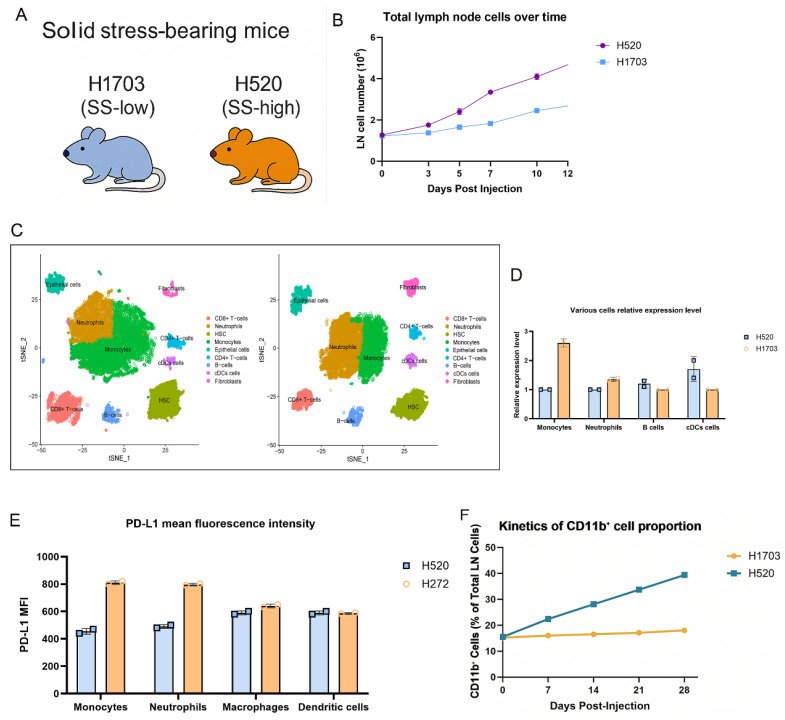
** Solid stress promotes lymph node expansion and immunosuppressive myeloid priming in pre-metastatic lung squamous carcinoma.** (A) Schematic of the syngeneic lung squamous cell carcinoma model. H520 (SS-high) or H1703 (SS-low) cells were injected subcutaneously, and draining lymph nodes were collected at indicated timepoints. (B) Total lymph node cellularity was significantly increased in H520-bearing mice compared to H1703. (C) UMAP projection of single-cell RNA-seq from LN immune cells showing increased infiltration of Ly6C^^hi^ monocytes and Ly6G⁺ neutrophils in H520 group. Left: H520 group, Right: H1703 group. (D) Flow cytometry quantification of various cell subsets. (E) PD-L1 mean fluorescence intensity (MFI) on monocytes, neutrophils, macrophages, and dendritic cells. (F) Kinetics of CD11b⁺ cell proportion in lymph nodes post-injection.

To investigate how solid stress (SS) affects the pre-metastatic lymph node (LN) niche, we subcutaneously implanted SS-high (H520) or SS-low (H1703) lung squamous carcinoma cells into immunocompetent mice (Fig. 1A). H520-bearing mice exhibited progressive enlargement of the axillary and brachial LNs. Notably, no tumor infiltration was detected in these LNs, suggesting that nodal remodeling occurred before metastatic colonization. Consistent with this observation, LN cellularity was significantly increased in the H520 group from day 7 onward (Fig. 1B), indicating SS-associated nodal hyperplasia. Single-cell RNA sequencing with UMAP revealed enrichment of CD11b⁺Ly6C^^hi^ monocytes and Ly6G⁺ neutrophils in H520-draining LNs, with reduced B cells and cDCs (Fig. 1C), confirmed by flow cytometry counts (Fig. 1D). PD-L1 expression was elevated on monocytes and neutrophils under high SS, as shown by increased mean fluorescence intensity (MFI) (Fig. 1E), implying heightened immunoregulatory potential. Longitudinal analysis revealed a time-dependent accumulation of CD11b⁺ cells preceding metastasis (Fig. 1F). These findings suggest that tumor-derived solid stress systemically reprograms nodal myeloid immunity, establishing a pre-metastatic niche.

### Solid stress induces spatially localized expansion of bone marrow-derived myeloid cells in tumor-draining lymph nodes

**Figure 2:**
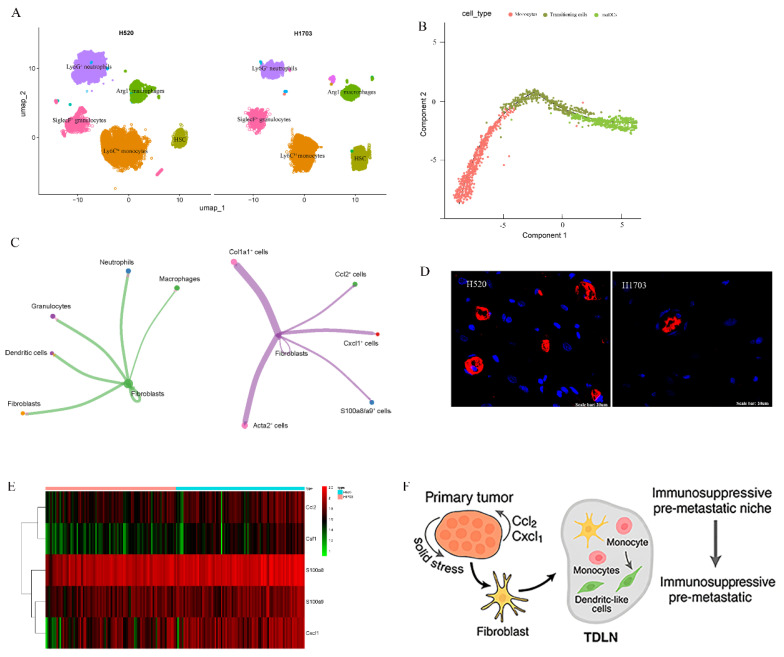
** Solid stress induces spatially localized expansion of bone marrow-derived myeloid cells in tumor-draining lymph nodes.** (A) UMAP of single-cell RNA-seq from TDLNs revealing distinct CD11b⁺Ly6C^hi, Ly6G⁺, and Arg1⁺ myeloid clusters enriched in H520 and H1703 group. (B) Pseudotime trajectory showing Ly6C^hi monocyte differentiation towards dendritic-like phenotypes under high-stress conditions. (C) Cell communication mapping of myeloid markers (S100a8, CXCL1) and stromal ligands (Ccl2, Acta2) across TDLNs from H520 mice. (D) Accumulation of GFP⁺CD11b⁺ cells in H520- and H1703-draining LNs. (E) Heatmap of chemokine expression (Ccl2, S100a8, Csf1) in H520-derived TDLNs across spatial zones. (F) Proposed model: Solid stress in primary tumors activates stromal-myeloid axis in TDLNs, leading to immunosuppressive pre-metastatic niche formation.

To examine how primary tumor solid stress (SS) reprograms the myeloid landscape in tumor-draining lymph nodes (TDLNs), we combined single-cell RNA-seq, spatial transcriptomics, and bone marrow (BM) tracing. ScRNA-seq of H520-TDLNs revealed marked enrichment of CD11b⁺ myeloid subsets, Ly6C^^hi^ monocytes, Ly6G⁺ neutrophils, SiglecF⁺ granulocytes, and Arg1⁺ macrophages, compared to H1703 (Fig. 2A). Pseudotime analysis showed Ly6C^hi monocytes transitioning into CD11c⁺MHC-II⁺ moDCs (Fig. 2B). Spatial transcriptomics revealed that these myeloid cells localized to specific regions, with CCL2, CXCL1, and S100a8 expressing cells co-localizing with fibroblasts, suggesting a myeloid-recruiting niche in the tumor microenvironment (Fig. 2C). Fibroblast markers Col1a1 and Acta2 were present in these regions, supporting a fibroblast-myeloid axis. Using GFP⁺ BM chimeras, we confirmed accumulation of GFP⁺CD11b⁺ cells in H520-draining LNs (Fig. 2D), indicating bone marrow origin. Chemokine heatmaps showed upregulation of Ccl2, Csf1, and S100a8 in H520-TDLNs (Fig. 2E), reinforcing a model where solid stress-activated fibroblasts guide BM-derived myeloid cell recruitment and positioning. These features define a biomechanically primed immunosuppressive niche (Fig. 2F).

### Solid stress promotes S100a8⁺ inflammatory remodeling of lymph nodes via fibroblast-myeloid crosstalk

**Figure 3:**
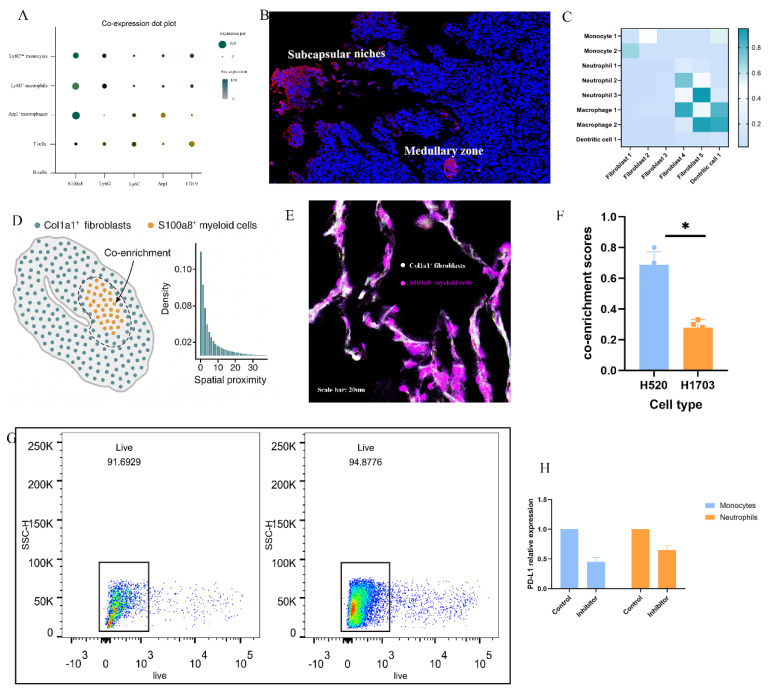
** Solid stress promotes S100a8⁺ inflammatory remodeling of lymph nodes via fibroblast-myeloid crosstalk.** (A) UMAP projection of single-cell RNA sequencing data showing enriched expression of *S100a8* across Ly6C^^hi^ monocytes, Ly6G⁺ neutrophils, and Arg1⁺ macrophages within H520-derived tumor-draining lymph nodes (TDLNs). (B) Spatial transcriptomic maps of *S100a8*, *CXCL1*, *CCL2*, and *CSF1* across representative H520-TDLN sections, highlighting co-localization within medullary and subcapsular regions. (C) Cell-cell heatmap analysis showing enriched fibroblast-myeloid signaling pathways, including *CCL2-CCR2* and *CSF1-CSF1R* interactions under high-stress conditions. (D) Quantitative spatial proximity analysis of Col1a1⁺ fibroblasts and S100a8⁺ myeloid cells, demonstrating strong co-enrichment within stress-activated stromal domains. (E) Representative immunofluorescence images showing co-localization of Col1a1 (white) and S100a8 (red) within TDLNs from H520-bearing mice, confirming fibroblast-myeloid interaction niches. Scale bar = 20 μm. (F) Quantification of Col1a1-S100a8 overlap index across multiple LN sections. Bars represent mean ± SEM. *P* < 0.05. (G) Flow cytometric gating and quantification of CD11b⁺S100a8⁺ myeloid cells in control vs. CCR2-inhibited H520-bearing mice, showing reduced myeloid accumulation upon blockade. Statistical significance was assessed using unpaired two-tailed t-test, P < 0.01. (H) Representative PD-L1 histograms of CD11b⁺ myeloid cells showing diminished expression following CCR2 inhibition, consistent with reduced immunosuppressive programming.

To elucidate how solid stress (SS) reprograms the tumor-draining lymph node (TDLN) microenvironment, we performed integrative analyses combining scRNA-seq, spatial transcriptomics, and functional validation. S100a8 was identified as a key inflammatory marker enriched in Ly6C^^hi^ monocytes, Ly6G⁺ neutrophils, and Arg1⁺ macrophages (Fig. 3A), with immunofluorescence results showing its localization to inflamed medullary and subcapsular niches alongside CCL2 and CXCL1 (Fig. 3B). Ligand-receptor modeling reveals partitioned signaling: Fibroblast_1 drives early monocyte recruitment via the CCL2-CCR2 axis, while activated Fibroblast_4 supports macrophage and neutrophil persistence through robust CSF1-CSF1R signaling (Fig. 3C). Spatial proximity and immunofluorescence confirmed co-localization between Col1a1⁺ fibroblasts and S100a8⁺ myeloid cells in H520-TDLNs (Fig. 3D-E), with significantly elevated co-enrichment scores (Fig. 3F). Pharmacologic inhibition of CCR2 reduced CD11b⁺S100a8⁺ cell accumulation (Fig. 3G) and decreased PD-L1 expression on monocytes and neutrophils (Fig. 3H), indicating that SS-induced fibroblast-myeloid circuits actively promote immunosuppressive niche formation in TDLNs.

### Solid stress-induced fibroblast-myeloid crosstalk promotes immunosuppressive myeloid differentiation and metastatic permissiveness

**Figure 4:**
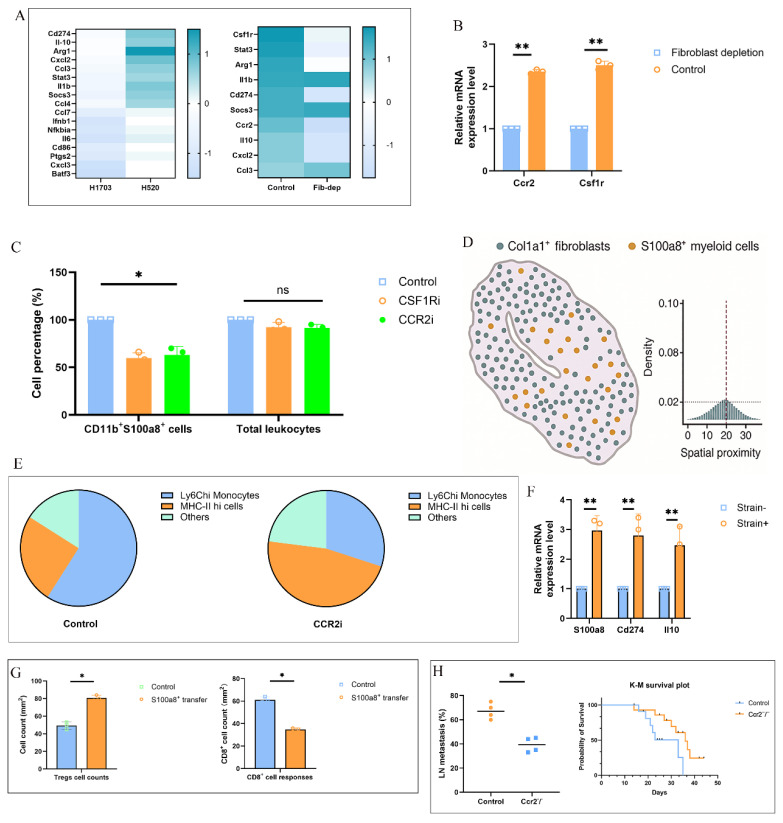
** Solid stress-induced fibroblast-myeloid crosstalk promotes immunosuppressive myeloid differentiation and metastatic permissiveness.** (A) Heatmap shows differentially expressed genes enriched for immunoregulatory programs including Cd274, Il10, and Arg1. (B) qPCR analysis of Ccr2 and Csf1r mRNA levels in CD11b⁺ myeloid cells isolated from TDLNs of control vs. fibroblast-ablated mice. Fibroblast depletion reduced chemokine receptor expression. Data represent mean ± s.e.m.; P < 0.05. (C) Flow cytometric quantification of CD11b⁺S100a8⁺ myeloid cells in TDLNs following CCR2 or CSF1R blockade. Frequencies were normalized to total CD45⁺ cells. (D) Spatial distribution analysis showing Col1a1, S100a8 in TDLNs. Disrupted fibroblast-myeloid co-enrichment was shown after pharmacologic inhibition of CCR2 or CSF1R. (E) Single-cell UMAP plots of CD11b⁺ populations in TDLNs with or without CCR2 inhibition. Pie charts represent altered cluster distribution, highlighting depletion of Ly6C^^hi^ monocytes and increase in MHC-II^^hi^ states. (F) *Ex vivo* co-culture of bone marrow-derived monocytes with LN fibroblasts subjected to cyclic mechanical strain (5%, 0.5 Hz). Monocyte mRNA expression of S100a8, Cd274, and Il10 measured by qPCR. (G) Quantification of draining LNs from naïve mice 48 h after adoptive transfer of sorted S100a8⁺ vs. control group. (H) Frequency of LN metastasis and Kaplan-Meier survival of H520 tumor-bearing wild-type vs. Ccr2⁻/⁻ mice. Left: incidence of metastatic lesions in axillary nodes. Right: median survival (WT: 21 days; KO: 37 days); log-rank test P < 0.01.

To assess whether solid stress-induced fibroblast-myeloid interactions directly mediate immunosuppression and metastasis, we performed mechanistic and functional assays. Bulk RNA-seq of sorted S100a8⁺ myeloid cells from H520-TDLNs revealed enrichment of Cd274, Il10, and Arg1, supporting an immunosuppressive phenotype (Fig. 4A). This transcriptional program was diminished upon fibroblast depletion, which also reduced Ccr2 and Csf1r expression in CD11b⁺ cells (Fig. 4B). Pharmacologic inhibition of CCR2 or CSF1R significantly decreased CD11b⁺S100a8⁺ cells without altering total leukocyte percentage (Fig. 4C), and multiplex analysis showed disrupted fibroblast-myeloid co-enrichment (Fig. 4D). Single-cell RNA-seq following CCR2 blockade revealed loss of Ly6C^^hi^ monocytes and skewing toward MHC-II^^hi^ states (Fig. 4E). In *ex vivo* co-culture, strained fibroblasts induced higher S100a8, Cd274, and Il10 in monocytes, confirming mechanical dependency (Fig. 4F). Adoptive transfer of S100a8⁺ monocytes enhanced Treg infiltration and impaired CD8⁺ cell responses in recipient mice (Fig. 4G). Finally, Ccr2⁻/⁻ mice bearing H520 tumors showed reduced LN metastasis and prolonged survival (Fig. 4H), demonstrating that fibroblast-myeloid circuits downstream of solid stress promote pre-metastatic immunosuppression.

### Solid stress activates fibroblast-STAT3 and YAP signaling to orchestrate a transcriptional program of immunosuppressive niche formation

**Figure 5:**
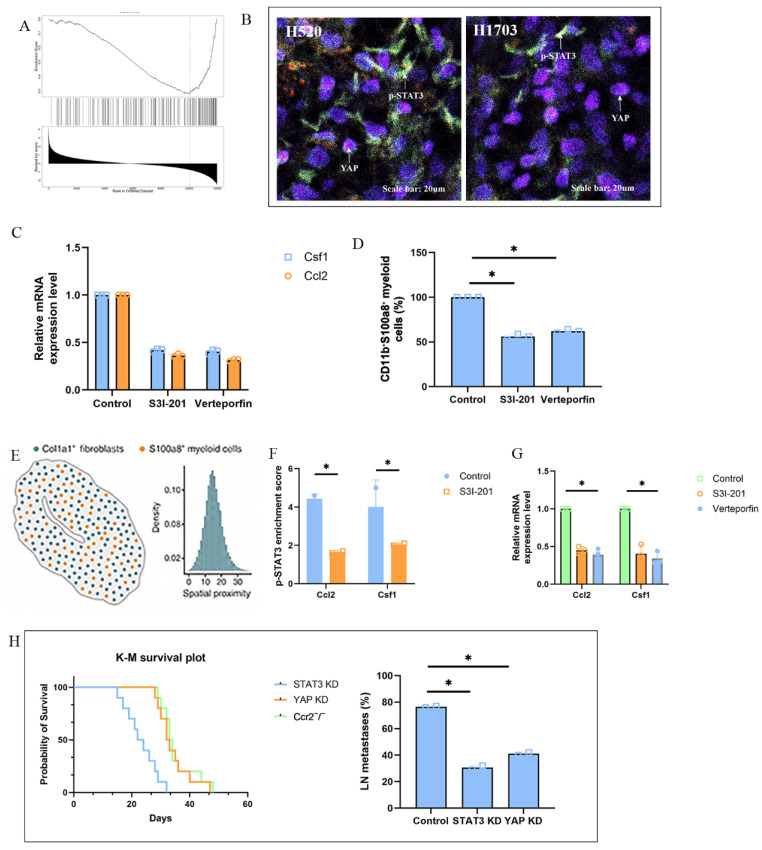
** Solid stress activates fibroblast-STAT3 and YAP signaling to regulate immunosuppressive niche formation.** (A) GSEA of fibroblasts from H520-TDLNs vs H1703-TDLNs showing enrichment of mechanoresponsive transcriptional programs, including STAT3, YAP/TAZ, and NF-κB pathways. (B) Representative immunofluorescence images showing nuclear localization of p-STAT3 and YAP in Col1a1⁺ fibroblasts in H520-TDLNs. Scale bar = 20 μm. (C) qPCR analysis of Ccl2 and Csf1 in CD90⁺ fibroblasts from H520-TDLNs treated with S3I-201, or Verteporfin and control group. (D) Flow cytometric quantification of CD11b⁺S100a8⁺ myeloid cells in TDLNs after STAT3 or YAP inhibition. (E) Spatial transcriptomic maps of H520-TDLNs showing reduced co-localization between Col1a1⁺ fibroblasts and S100a8⁺ myeloid cells after inhibition. Disrupted fibroblast-myeloid co-enrichment was shown after pharmacologic inhibition. (F) ChIP-qPCR of p-STAT3 and YAP binding at Ccl2 and Csf1 promoters in primary fibroblasts under control vs strained conditions. (G) qPCR of Ccl2 and Csf1 expression after STAT3 or YAP inhibition. (H) Kaplan-Meier survival curves and frequency of axillary LN metastasis in H520-bearing mice after knockdown STAT3 and YAP.

To dissect how solid stress translates into immunosuppressive microenvironmental programming, we focused on fibroblast-intrinsic mechanotransduction pathways. Gene set enrichment analysis (GSEA) of fibroblasts isolated from H520-TDLNs revealed upregulation of mechanoresponsive transcriptional programs including STAT3, YAP/TAZ, and NF-κB signatures compared to fibroblasts from low-stress H1703-TDLNs (Fig. 5A). Immunofluorescence analysis confirmed increased nuclear p-STAT3 and YAP localization in Col1a1⁺ fibroblasts within inflamed stromal regions (Fig. 5B), particularly in proximity to S100a8⁺ myeloid clusters. To assess the functional role of these pathways, we treated H520-bearing mice with the STAT3 inhibitor S3I-201 or the YAP-TEAD interaction blocker Verteporfin. Both treatments led to a marked decrease in stromal Ccl2 and Csf1 transcript levels, accompanied by reduced accumulation of CD11b⁺S100a8⁺ myeloid cells (Fig. 5C-D). Spatial transcriptomic profiling showed that inhibition of either pathway abrogated the fibroblast-myeloid co-localization signature, with diminished overlap between Col1a1⁺ and S100a8⁺ signals and reduced expression of inflammatory chemokines in medullary and subcapsular zones (Fig. 5E). Mechanistically, chromatin immunoprecipitation (ChIP) of LN fibroblasts demonstrated enriched binding of p-STAT3 and YAP at the Ccl2 and Csf1 promoter/enhancer regions under high-strain conditions, which was abrogated by pharmacologic inhibition (Fig. 5F). Notably, cyclic mechanical strain (5%, 0.5 Hz) applied to primary LN fibroblasts *in vitro* resulted in synergistic nuclear co-localization of p-STAT3 and YAP, and inhibition of either factor abolished strain-induced upregulation of Ccl2 and Csf1 (Fig. 5G). Finally, knockdown of STAT3 or YAP in H520 tumor-bearing mice significantly delayed LN metastasis and extended survival (Fig. 5H). These findings identify a solid stress-responsive STAT3/YAP transcriptional program in fibroblasts that promotes immunoregulatory chemokine production and drives myeloid-dependent metastatic niche formation.

### Pharmacological disruption of the fibroblast-STAT3/YAP-myeloid axis reverses immunosuppressive niche programming and attenuates lymph node metastasis

**Figure 6:**
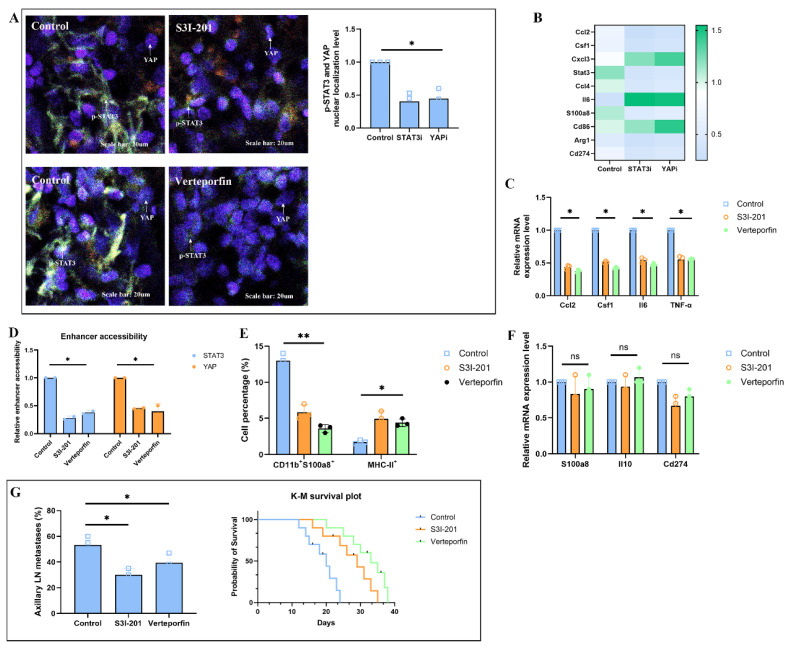
** Pharmacological inhibition of the fibroblast-STAT3/YAP-myeloid axis reduces lymph node metastasis.** (A) Immunofluorescence of H520-TDLNs showing reduced p-STAT3 and YAP nuclear localization in Col1a1⁺ fibroblasts and diminished co-localization with S100a8⁺ myeloid cells after STAT3 or YAP inhibition. Scale bar = 20 μm. (B) Heatmap showing decreased Ccl2 and Csf1 expression after the use of S3I-201, or Verteporfin compared to control group. (C) qPCR of Ccl2, Csf1, IL6, and TNF-α in CD90⁺ fibroblasts from H520-TDLNs after treatment with S3I-201, or Verteporfin and control group. (D) ATAC-seq showing reduced chromatin accessibility at STAT3 and TEAD enhancer regions near Ccl2 and Csf1 loci in fibroblasts from treated mice. (E) Flow cytometry quantification of CD11b⁺S100a8⁺ myeloid cells and MHC-II⁺ antigen-presenting subsets in TDLNs after treatment. (F) qPCR of S100a8, Il10, and Cd274 in BM-derived monocytes cultured with conditioned media from strained fibroblasts treated with inhibitors. (G) Frequency of axillary LN metastasis and Kaplan-Meier survival curves in H520-bearing mice treated with S3I-201, or Verteporfin and control group.

Multiplex immunofluorescence revealed that treatment with either S3I-201 (STAT3 inhibitor) or Verteporfin (YAP-TEAD inhibitor) significantly reduced nuclear localization of p-STAT3 and YAP in Col1a1⁺ fibroblasts, with concomitant decreases in spatial overlap between fibroblast and S100a8⁺ myeloid cell compartments (Fig. 6A). Spatial transcriptomic mapping further confirmed the loss of fibroblast-myeloid co-enrichment signatures, particularly in medullary and subcapsular regions, alongside marked reductions in Ccl2 and Csf1 expression (Fig. 6B). At the gene level, sorted CD90⁺ fibroblasts from treated TDLNs exhibited downregulation of mechanoresponsive and immunomodulatory genes, including Ccl2, Csf1, Il6, and TNF-α, as measured by qPCR (Fig. 6C). Chromatin accessibility profiling by ATAC-seq revealed a collapse of enhancer accessibility at STAT3 and YAP-TEAD bound regions near these loci (Fig. 6D). Immunophenotypic analysis by flow cytometry demonstrated a rebalancing of the myeloid compartment, with significant reductions in CD11b⁺S100a8⁺ suppressive subsets and restoration of MHC-II⁺ antigen-presenting cell populations (Fig. 6E). Functional *ex vivo* assays showed that conditioned media from inhibitor-treated fibroblasts failed to induce S100a8, Il10, or Cd274 expression in naïve monocytes, confirming the disruption of paracrine education (Fig. 6F). Functionally, treated mice exhibited markedly reduced incidence of axillary LN metastases, as detected by cytokeratin⁺ immunohistochemistry, and showed significant prolongation of survival (Fig. 6G). Together, these findings demonstrate that pharmacological inhibition of the fibroblast-STAT3/YAP-myeloid axis reverses solid stress-induced immunosuppressive conditioning within the TDLN, thereby attenuating metastatic progression. These results position stromal mechanosensing pathways as actionable therapeutic targets in the prevention of lymphatic dissemination.

### Solid stress-induced fibroblast-myeloid crosstalk restricts cytotoxic T cell infiltration and function in pre-metastatic lymph nodes

**Figure 7:**
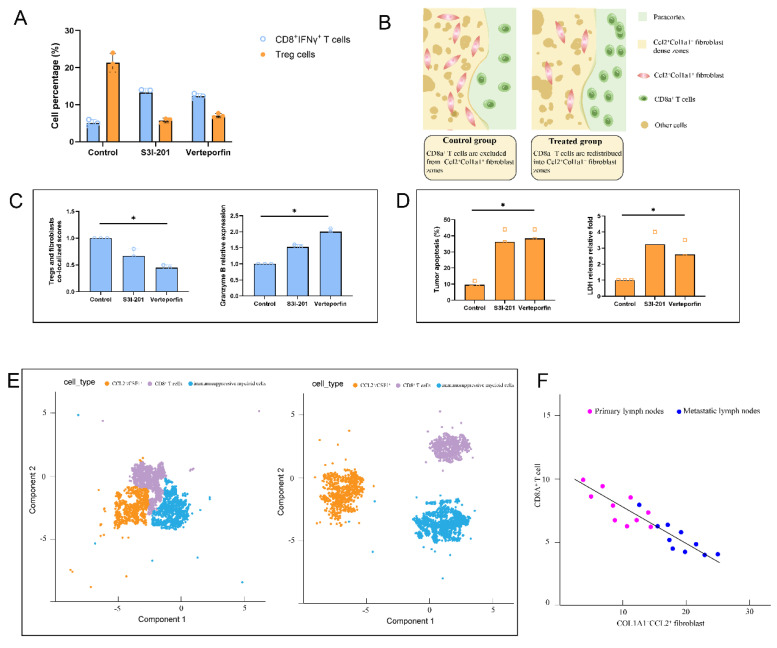
** Solid stress-induced fibroblast-myeloid crosstalk restricts CD8⁺ T cell infiltration and function in tumor-draining lymph nodes.** (A) Flow cytometric quantification of CD8⁺IFNγ⁺ effector T cells and Foxp3⁺ regulatory T cells (Tregs) in TDLNs from H520-bearing mice treated with S3I-201, or Verteporfin. CD8⁺/Treg ratios are shown; values normalized to CD45⁺ leukocytes. (B) Spatial transcriptomic data showing the localization of CD8A⁺ and FOXP3⁺ T cells relative to Col1a1⁺ fibroblasts and chemokine-expressing zones (CCL2, CSF1) in TDLNs following treatment. (C) Quantification of Tregs and fibroblasts co-localized scores and Granzyme B and in TDLNs showing enhanced cytotoxic T cell activity upon STAT3/YAP inhibition. (D) Tumor-killing capacity of CD8⁺ T cells isolated from TDLNs assessed in autologous tumor spheroid co-culture. Apoptotic index and LDH release measured in triplicate. (E) Single-cell RNA-seq analysis of TDLNs showing CCL2⁺/CSF1⁺ fibroblast clusters, immunosuppressive myeloid cells, and exhausted CD8⁺ T cells co-enriched in control group (left). Disruption of tri-cellular niche observed with S3I-201 treatment (right). (F) Cross-species spatial transcriptomic correlation in human LUSC samples showing inverse association between Col1a1^+^ fibroblast abundance and CD8A⁺ T cell infiltration in both primary tumors and metastatic lymph nodes (*R²* = -0.64, *P* < 0.01).

To evaluate whether solid stress-primed fibroblasts indirectly impair cytotoxic T cell-mediated immunity in the pre-metastatic niche, we first profiled T cell phenotypes in tumor-draining lymph nodes (TDLNs) of H520-bearing mice. Flow cytometry revealed a significant reduction of CD8⁺IFNγ⁺ cytotoxic T cells and an increase in Foxp3⁺ regulatory T cells (Tregs) in the control group, while both S3I-201 and Verteporfin treatment restored CD8⁺ effector cell frequencies and reduced Tregs cells, resulting in a markedly increased CD8⁺/Treg ratio (Fig. 7A). Spatial transcriptomic data were visualized using AI graphic generation tools further showed exclusion of CD8A⁺ T cells from Ccl2⁺Col1a1⁺ fibroblast-dense zones in control group nodes, whereas treatment led to their redistribution into paracortical regions (Fig. 7B). Immunohistochemical results confirmed enhanced Granzyme B expression in TDLNs from S3I-201 or Verteporfin-treated mice, supporting the restoration of T cell cytotoxic activity (Fig. 7C). T cells from the S3I-201 or Verteporfin groups exhibited significantly increased tumor-killing capacity, as assessed by tumor apoptosis and LDH release (Fig. 7D). Single-cell RNA sequencing revealed that fibroblasts in control TDLNs exhibited a CCL2⁺/CSF1⁺ inflammatory phenotype, accompanied by enrichment of exhausted CD8⁺ T cells and immunosuppressive myeloid cells. Upon treatment, this immunosuppressive tri-cellular niche was disrupted, as shown by reduced spatial and transcriptomic overlap (Fig. 7E). Finally, cross-species analysis of human lung squamous cell carcinoma (LUSC) spatial transcriptomic data revealed a significant inverse correlation between Col1a1⁺ fibroblast abundance and CD8A⁺ T cell infiltration in both primary tumors and metastatic lymph nodes (Fig. 7F). Collectively, these findings demonstrate that solid stress-primed fibroblast-myeloid interactions form a T cell-exclusionary immunosuppressive niche in lymph nodes, which can be reversed via pharmacologic disruption of STAT3/YAP signaling.

## Discussion

This study reveals a previously unrecognized role of tumor-derived solid stress in shaping the pre-metastatic microenvironment of lung squamous cell carcinoma (LUSC). Through a combination of murine models, single-cell and spatial transcriptomics, and mechanistic perturbation assays, we demonstrate that mechanical stress generated during tumor expansion drives fibroblast activation and establishes a fibroblast-myeloid signaling circuit that remodels tumor-draining lymph nodes (TDLNs) before overt metastasis. This process promotes the accumulation of S100a8⁺ immunosuppressive myeloid cells, suppresses cytotoxic T cell function, and facilitates metastatic permissiveness.

A defining feature of our findings is the identification of solid stress as a systemic regulator that extends beyond the primary tumor. Mice bearing high-stress tumors exhibited early enlargement and immune remodeling of draining lymph nodes in the absence of detectable metastatic cells, indicating that mechanical cues alone can precondition distant lymphatic sites. Single-cell analyses further revealed that this remodeling is characterized by an expansion of CD11b⁺Ly6C^^hi^ and Ly6G⁺ myeloid populations, accompanied by elevated PD-L1 expression and increased S100a8 levels. These observations are consistent with prior reports describing S100a8⁺ myeloid cells as mediators of inflammatory niche formation 19-20, yet our work links their emergence to a mechanical origin, a concept that has not been previously addressed in LUSC.

Mechanistically, fibroblasts within TDLNs act as key mediators of stress-induced immunomodulation 21-23. Spatial transcriptomics identified Col1a1⁺ fibroblasts that co-localize with S100a8⁺ myeloid clusters and exhibit high expression of Ccl2 and Csf1, suggesting a fibroblast-driven recruitment and activation of immunoregulatory myeloid cells. Functional inhibition experiments confirmed this hypothesis: blocking CCR2 or CSF1R signaling reduced S100a8⁺ myeloid infiltration and partially restored antigen-presenting cell populations. These results establish fibroblast-derived CCL2/CSF1 as essential components of the stress-induced inflammatory axis 24.

We further demonstrate that the activation of fibroblasts under solid stress depends on the STAT3 and YAP signaling pathways. Both transcription factors exhibited nuclear localization in stressed fibroblasts, and their inhibition markedly reduced chemokine expression and disrupted fibroblast-myeloid co-localization, these findings are consistent with previous studies 25-26. Chromatin accessibility and ChIP-qPCR analyses revealed that STAT3 and YAP jointly bind enhancer regions near Ccl2 and Csf1, indicating a direct transcriptional mechanism 27-29. Pharmacological inhibition of either pathway reversed lymph node immunosuppression and delayed metastasis, confirming the functional relevance of this mechanotransduction module. These findings extend current knowledge by linking biomechanical strain to fibroblast-driven immune regulation and metastasis 30-31.

From an immunological standpoint, this fibroblast-myeloid axis creates a nodal environment that favors immune tolerance 32-33. The expansion of S100a8⁺ myeloid cells coincided with depletion of CD8⁺ effector T cells and enrichment of regulatory T cells 34. Blocking STAT3 or YAP signaling restored CD8⁺ T cell infiltration, enhanced cytotoxic activity, and improved survival outcomes 35-36. Using human LUSC spatial datasets further confirmed the inverse relationship between Col1a1⁺ fibroblast density and CD8A⁺ T cell presence in both primary tumors and metastatic nodes, suggesting that this pathway is conserved in human disease.

While these findings provide a mechanistic framework linking mechanical forces to lymphatic immune remodeling, several limitations should be acknowledged. The use of subcutaneous implantation, although suitable for controlled analysis of stress-dependent effects, may not fully replicate native lung biomechanics. While the subcutaneous model has been instrumental in dissecting the role of solid stress in immune remodeling, it is important to recognize that the lymphatic drainage and the tumor microenvironment in subcutaneous tumors differ significantly from those in orthotopic tumors. Subcutaneous tumors primarily drain to axillary and brachial lymph nodes, which do not mimic the natural drainage pathways of lung tumors. In contrast, orthotopic tumors are directly implanted into the lung and drain to regional mediastinal lymph nodes, providing a more accurate representation of the metastatic niche. Therefore, findings from the subcutaneous model may not fully recapitulate the immune dynamics and metastatic processes observed in the orthotopic setting. In addition, while STAT3 and YAP were identified as dominant transcriptional mediators, other mechanosensitive pathways such as FAK or NF-κB signaling may also participate and warrant further exploration. Finally, validation of these mechanisms in human tissue cohorts will be essential to determine their prognostic and therapeutic significance.

In conclusion, this study establishes a direct mechanistic link between tumor-derived solid stress and immune remodeling within tumor-draining lymph nodes. By activating fibroblast STAT3/YAP signaling, mechanical forces drive chemokine-mediated recruitment of immunosuppressive myeloid cells and exclusion of cytotoxic T cells, thereby priming a permissive metastatic niche. However, as these findings are still at an early stage, further validation in preclinical models and human tissue cohorts is necessary before drawing clinical conclusions. Targeting this biomechanical-immunologic axis offers a promising strategy to prevent lymphatic dissemination and improve outcomes in patients with LUSC.

## Conclusion

Solid stress in LUSC tumors reprograms tumor-draining lymph nodes by activating fibroblasts to recruit immunosuppressive S100a8⁺ myeloid cells. This stromal-immune crosstalk establishes a pre-metastatic niche prior to tumor cell arrival. Targeting this mechano-immune axis may offer new strategies to prevent early lymphatic dissemination.

## Data Availability

The data that support the findings of this study are available from the corresponding author upon reasonable request.
